# Supplementary material for: Screening and Assessment of Hypoglycemic Active Peptide from Natural Edible Pigment Phycobiliprotein Based on Molecular Docking, Network Pharmacology, Enzyme Inhibition Assay Analyses, and Cell Experiments
Source: Mar Drugs. 2025 Aug 17;23(8):331. doi: 10.3390/md23080331 (PMC12387383; doi:10.3390/md23080331)
Supplement: Supplementary file 1 [file marinedrugs-23-00331-s001.zip › marinedrugs-3790728-supplementary.pdf]

Supplementary materials

Table S1. The identified peptides sequences and docking results of *Arthrospira platensis* phycobiliproteins peptide extracts.

Peptide Sequence	3wy 1	4a5 s	Peptide Sequence	3wy 1	4a5 s
QGRFL	-7.5	-8.3	YYLRY	-7.1	-9.1
VAFGRF	-8	-9.4	FELSPSWYIEALK	-5.4	-7.2
SQGRFL	-6.8	-8.5	VGADAGKEMGIYF	-7.2	-8.1
QVAFGRF	-7	-9.1	LAGDPSILDERVLNGLK	-5.4	-6.8
IGYYLRM	-7.5	-8.4	YNKFPYT	-7.6	-7.9
ARDIGYYLRM	-6.7	-6.9	LAGDPSILDERVLNGLKETYNS L	-5.2	-6.9
IQVAFGRF	-7.6	-9.2	LRDLDYYLR	-7.1	-8.2
SYFDR	-5.8	-8.2	AVYNKFPY	-6.2	-8.2
DSQGRFL	-7.2	-8.7	PAGITPGDCSAL	-7.1	-8.1
DLDYYLRL	-5.8	-7.6	IRDLDYYLR	-6.7	-8.6
ASYFDR	-7.7	-7.2	EDRCLNGL	-6.6	-8.2
LDYYLRL	-6.6	-8.1	RDLDYYL	-7.8	-7.4
SPSWYIEALK	-4.7	-8.1	VYNKFPY	-6.8	-8.2
GDPSILDERVLNGLK	-5.4	-7.7	GDPSILDERVL	-6.2	-8
AGDASVLEDRCLNGL	-5.7	-7.6	ARSLFA	-8.2	-7.6
YNKFPY	-7.3	-8.5	AGDPSILDERVL	-6.5	-6.9
MFDAFTK	-5	-8.2	NKFPYT	-6.2	-8.9
DYYLRL	-7.4	-8.4	GRFRQAKAGL	-7.3	-8.6
AARSLF	-7.3	-7.7	YAACIR	-7.5	-7.8
GYYLR	-8	-8.9	AVYNKFPYT	-6.2	-8.2
DIGYYLR	-7.7	-8.2	GSSVAVGVGKMK	-5.5	-7.2

ASYFDRA	-8.2	-8.2	NAARSLF	-7	-8.4
MAACLR	-6.5	-7.8	ELSPSWYIEALK	-6.3	-7.8
KFPYT	-6.2	-7.9	VSIADSQGRFL	-7.1	-7.4
AGDPSILDERVLNGLK	-5.9	-6.4	AVSIADSQGRFL	-6.5	-8.4
NKFPY	-7.1	-8.7	SPGELDRIKSF	-5.8	-6.8
YYLRL	-7.4	-8.9	NKFPYTT	-7.7	-9.1
PSWYIEALK	-6.3	-8.2	RYVTY	-8.2	-8.3
SYFDRA	-8	-8.5	EINRTFE	-7.9	-7.9
DLDYYLR	-5.7	-6.9	RPDVVSP	-8.2	-8
EIQVAFGRF	-5.4	-7.7	SIVNADAEAR	-7.6	-7.8
LSPSWYIEALK	-5.7	-7.1	RDLDY	-7.8	-8
IGYYLR	-7.7	-8.2	LRDLDY	-7.9	-8.7
FDAFTK	-7.7	-8.9	LRYVT	-7.5	-8.3
SIADSQGRFL	-6.6	-8.1	RTFEL	-7.8	-8.3
IADSQGRFL	-6.4	-8.2	IRDLDY	-7.8	-7.8
IASYFDR	-8.2	-8.9	IRDLDYY	-7.8	-8.9
LDYYLRY	-6.8	-8.6	LRDLDYY	-7.7	-8.6
SIADSQGRF	-7.8	-8.6	RTFELSPSW	-6.0	-8.8
LDYYLR	-8.2	-8	DAVNRITS	-7.6	-7.7
DYYLRY	-7.0	-9.3	STEIQVAFGRFRQ	-6.0	-7.5
YFDRA	-6.8	-8.9	LAGDPSILDERV	-8.0	-7.3
RDLDYYLR	-6.6	-8.9	NAARSLFA	-8	-8
IADSQGRF	-7.3	-7.9	KSIVNADAEARY	-7.8	-7.4
DYYLR	-7.5	-9	LSSTEIQVAFGRFRQ	-7.5	-6.7
IASYFDRA	-6.7	-8.9	IADSQGRFLS	-7.6	-9

Table S2. The databases and platform websites in this procedure.

**Database**	**Website**
NCBI	http://www.ncbi.nlm.nih.gov/protein
NovoPro	https://www.novopro.cn/tools/calc_peptide_property.html
BIOPEP-UWM	https://biochemia.uwm.edu.pl/biopep-uwm
PeptideRanker	http://distilldeep.ucd.ie/PeptideRanker
AlgPred	https://webs.iiitd.edu.in/raghava/algpred
Innovagen	https://pepcalc.com
admetSAR	http://lmmd.ecust.edu.cn/admetsar1/predict
ToxinPred	https://webs.iiitd.edu.in/raghava/toxinpred/index.html
SwissADME	http://www.swissadme.ch
PDB	https://www.rcsb.org
SwissTargetPrediction	http://www.swisstargetprediction.ch
GeneCards	https://www.genecards.org
OMIM-GENE-MAP	https://www.omim.org/search/advanced/geneMap
TTD	http://www.bidd.nus.edu.sg/group/cjttd
Venny 2.1	https://bioinfogp.cnb.csic.es/tools/venny/index.html
STRING	http://string-db.org
Cytoscape 3.9.1	http://www.cytoscape.org
Metscape	https://metascape.org/gp/index.html
Bioinformatics	http://www.bioinformatics.com.cn
